# Foam Cell Induction Activates AMPK But Uncouples Its Regulation of Autophagy and Lysosomal Homeostasis

**DOI:** 10.3390/ijms21239033

**Published:** 2020-11-27

**Authors:** Nicholas D. LeBlond, Julia R. C. Nunes, Tyler K. T. Smith, Conor O’Dwyer, Sabrina Robichaud, Suresh Gadde, Marceline Côté, Bruce E. Kemp, Mireille Ouimet, Morgan D. Fullerton

**Affiliations:** 1 Department of Biochemistry, Microbiology and Immunology, Faculty of Medicine, University of Ottawa, Roger Guindon Hall, 451 Smyth Rd, Ottawa, ON K1H 8M5, Canada; nlebl062@uottawa.ca (N.D.L.); jroli011@uottawa.ca (J.R.C.N.); tsmit121@uottawa.ca (T.K.T.S.); codwyer@uottawa.ca (C.O.); srobi118@uottawa.ca (S.R.); marceline.cote@uottawa.ca (M.C.); mireille.ouimet@uottawa.ca (M.O.); 2 Centre for Infection, Immunity and Inflammation, Ottawa, ON K1H 8M5, Canada; sgadde@uottawa.ca; 3 Centre for Catalysis Research and Innovation, Ottawa, ON K1H 8M5, Canada; 4 University of Ottawa Heart Institute, Ottawa, ON K1Y 4W7, Canada; 5 Department of Cellular and Molecular Medicine, Faculty of Medicine, University of Ottawa, Ottawa, ON K1H 8M5, Canada; 6 St. Vincent’s Institute of Medical Research and Department of Medicine, University of Melbourne, Fitzroy, Melbourne, VIC 3065, Australia; bkemp@svi.edu.au; 7 Mary MacKillop Institute for Health Research, Australian Catholic University, Melbourne, VIC 3065, Australia

**Keywords:** macrophage, AMP-activated protein kinase (AMPK), autophagy, immunometabolism, lysosomal homeostasis, atherosclerosis, foam cell, lipids

## Abstract

The dysregulation of macrophage lipid metabolism drives atherosclerosis. AMP-activated protein kinase (AMPK) is a master regulator of cellular energetics and plays essential roles regulating macrophage lipid dynamics. Here, we investigated the consequences of atherogenic lipoprotein-induced foam cell formation on downstream immunometabolic signaling in primary mouse macrophages. A variety of atherogenic low-density lipoproteins (acetylated, oxidized, and aggregated forms) activated AMPK signaling in a manner that was in part due to CD36 and calcium-related signaling. In quiescent macrophages, basal AMPK signaling was crucial for maintaining markers of lysosomal homeostasis as well as levels of key components in the lysosomal expression and regulation network. Moreover, AMPK activation resulted in targeted upregulation of members of this network via transcription factor EB. However, in lipid-induced macrophage foam cells, neither basal AMPK signaling nor its activation affected lysosomal-associated programs. These results suggest that while the sum of AMPK signaling in cultured macrophages may be anti-atherogenic, atherosclerotic input dampens the regulatory capacity of AMPK signaling.

## 1. Introduction

Atherosclerosis precedes and predicts the development of cardiovascular disease, which is a leading cause of morbidity and mortality worldwide. There are numerous risk factors, however, elevated circulating cholesterol (hypercholesterolemia) is the main driver of atherosclerosis. In the early stages, cholesterol-rich low-density lipoproteins (LDL) accumulate in the subendothelial space where it is prone to modifications such as aggregation and oxidation [1,2,3,4]. Endothelial activation can subsequently invoke an inflammatory response that stimulates the upregulation of adhesion molecules and chemoattractant proteins that facilitates the recruitment and transmigration circulating monocytes [5,6]. Within the atherosclerotic microenvironment, monocyte-derived and/or proliferating macrophages play an important role by scavenging modified LDL particles, leading to foam cell formation and propagating a pro-inflammatory plaque milieu [7,8,9,10]. Modeling lipid dynamics in cultured macrophages, usually via loading with acetylated-LDL (acLDL), oxidized-LDL (oxLDL), or aggregated-LDL (agLDL), has been crucial to understanding the initiation and progression of atherosclerosis in differentiated macrophages.

AMP-activated protein kinase (AMPK) governs numerous pathways in lipid metabolism and inflammation. Acting as a critical gauge of cellular energy, AMPK inhibits lipid and protein synthesis, while stimulating autophagy and oxidative metabolism [11,12]. AMPK is activated in response to changes in cellular energy and glucose deprivation (via liver kinase B1; LKB1) as well as to increases in cytosolic calcium (via calmodulin-dependent protein kinase kinase-2; CaMKK2) [13,14,15,16,17]. We and others have linked AMPK to the regulation of macrophage mitochondrial content, fatty acid oxidation, and inflammatory signaling [18,19,20,21]. While the contribution of macrophage AMPK to atherosclerosis remains unresolved, AMPK signaling is critical for the differentiation of monocytes to macrophages as well as the removal of cholesterol from lipid-laden macrophages in culture and in vivo [22,23,24].

Macroautophagy (herein referred to as autophagy) is an evolutionarily conserved ‘self-eating’ process that plays an important role in the processing of lipid-droplet-associated cholesterol prior to its efflux and is important in atherosclerosis progression and regression [25,26]. AMPK is a positive regulator of autophagy and is known to both directly (via phosphorylation of Unc-like 51 kinase; ULK1 and many others) and indirectly (via the inhibition of mechanistic target of rapamycin complex 1; mTORC1) activate autophagy [27,28]. In addition, AMPK has been recently shown to directly promote transcription factor EB (TFEB) transcriptional activity, a master regulator of genes within autophagy and lysosomal biogenesis [29,30,31,32,33]. Interestingly, atherogenic lipids have been shown to both disrupt lysosomal function, while simultaneously stimulating lysosomal biosynthesis and autophagy via TFEB [34].

AMPK sits at a nexus of macrophage immunometabolism, however, how its activity and signaling may be altered upon in vitro foam cell formation remains unknown, but relevant in the context of atherogenesis. Here, we report that in response to various forms of modified LDL, AMPK was activated in a CD36- and CaMKK2-dependent manner. Macrophage AMPK signaling was necessary to maintain normal lysosomal function, but became overwhelmed and failed to maintain or stimulate these programs during foam cell induction.

## 2. Results

### 2.1. Atherogenic Lipids Activate AMPK in Bone Marrow-Derived Macrophages

**Figure 1:**
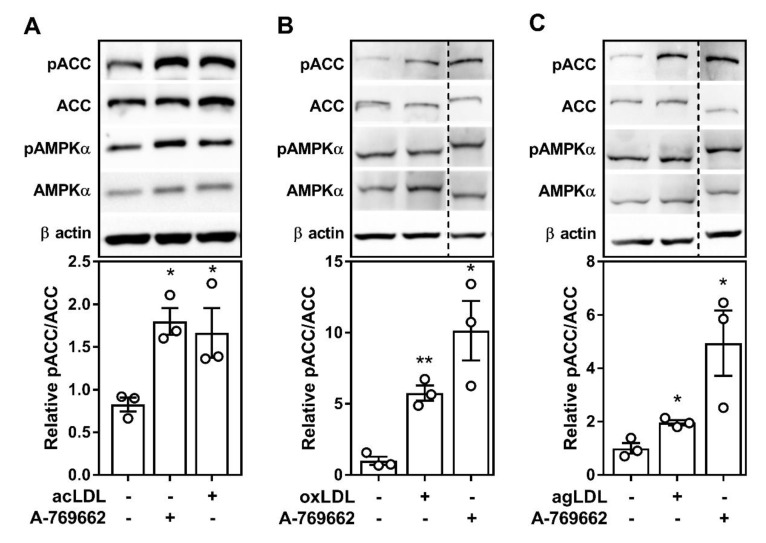
Atherogenic lipoproteins augment AMP-activated protein kinase (AMPK)-specific signaling in cultured macrophages. (**A**–**C**) Cells were isolated from wild-type (WT) mice, then incubated with either acetylated LDL (**A**; 50 µg/mL), oxidized LDL (**B**; 50 µg/mL), or aggregated LDL (**C**; 50 µg/mL) for 24 h (**A**) and 18 h (**B**,**C**). Cells were incubated with A-769662 (**A**–**C**; 100 µM) for 24 h (**A**) and 18 h (**B**,**C**) to serve as a positive control for AMPK activation. (**A**–**C**) Duplicate gels were used to assess total and phosphorylated proteins. Dashed line signifies where an image was cropped but represent the same gel. Phosphorylated acetyl Co-A carboxylase (ACC)/total ACC (260 kDa), pAMPKα/AMPKα (60 kDa), and β actin (37 kDa). Results are representative of three independent experiments and data represent the mean ± SEM, where * and ** represent a statistical difference of *p* < 0.05 and *p* < 0.01, respectively, compared to vehicle control.

We set out to determine the effect of atherogenic lipids on AMPK signaling in mouse primary bone marrow-derived macrophages (BMDM). Given the tendency to use acLDL to perform in vitro macrophage cholesterol experiments, we first assessed AMPK signaling with and without acLDL and observed a significant increase in the activating phosphorylation of AMPK and downstream signaling to a primary AMPK substrate, acetyl-CoA carboxylase (ACC) (Figure 1A). We next used the oxLDL and agLDL forms of LDL (Figure 1B,C). After the 8 and 24 h treatments, AMPK signaling remained active, although not to the same extent as pharmacological activation with the direct allosteric activator A-769662 (Supplementary Figure S1).

### 2.2. Atherogenic Lipoproteins Activate Macrophage AMPK Partially via CaMKK2

**Figure 2:**
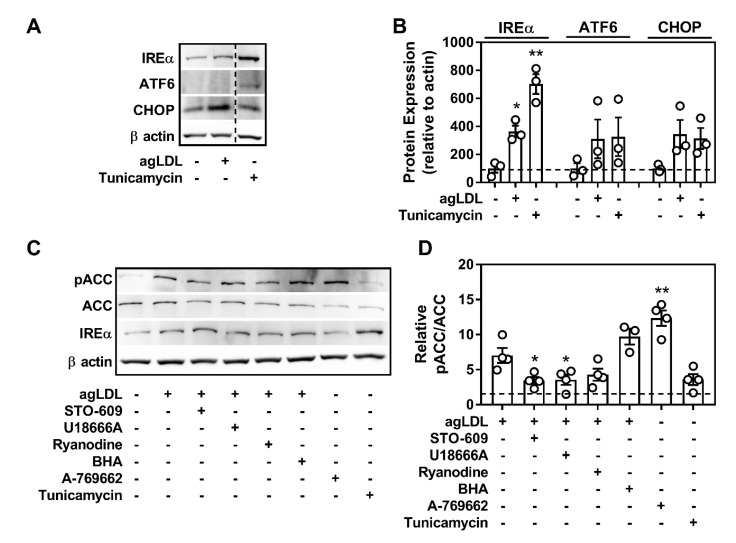
Aggregated (agLDL) induces endoplasmic reticulum (ER) stress and activates AMPK partially through calmodulin-dependent protein kinase kinase-2 (CaMKK2) signaling in cultured macrophages. (**A**) Representative immunoblots depicting the induction of ER stress by upregulation of its markers IREα (130 kDa), ATF6 (90 kDa), and CHOP (27 kDa) in response to agLDL (50 µg/mL). Dashed line in **A** signifies where the image was cropped but represents the same gel. (**B**) Relative quantification of ER stress markers relative to β actin. (**C**) Assessing the potential role that CaMKK2 (STO-609; 25 µM), cholesterol trafficking (U18666A; 1 µM), ER-calcium release (Ryanodine; 1 µM), and ROS (BHA; 100 µM) play a role in activating macrophage AMPK in response to agLDL (50 μg/mL). (**D**) Relative quantification of pACC to ACC signal, shown relative to the vehicle control treated (no agLDL) cells (dashed line). Macrophages isolated from WT mice were incubated with agLDL (50 µg/mL) along with inhibitors for 18 h. A-769662 (100 µM) and Tunicamycin (2.5 µg/mL) were used as a positive control for AMPK activation and ER stress, respectively. pACC/ACC (260 kDa) and β actin (37 kDa). Data are the mean ± SEM and are representative of at least three independent experiments using macrophages isolated from separate mice, where * and ** represent *p* < 0.05 and *p* < 0.01 compared to the vehicle control for (**A**) and compared to agLDL-treated cells in (**D**).

In the context of a plaque-resident, differentiated macrophage, the cause and effect of AMPK activation in response to atherogenic lipids was our next focus. The endoplasmic reticulum (ER) is important for maintaining cytosolic ionic calcium homeostasis and induction of ER stress can lead to increased cytosolic calcium [35]. Atherogenic lipids caused higher levels of ER stress in BMDM as seen by the increase in IREα, ATF6, and CHOP expression (Figure 2A,B for agLDL and Supplementary Figure S2A,B for oxLDL), which are known markers of ER stress that have been documented in various cell types involved in atherogenesis [3,36]. We hypothesized that uptake and processing of excess cholesterol from atherogenic lipids led to heightened ER stress and caused activation of AMPK by its upstream kinase, CaMKK2.

To address this, we co-cultured BMDM with atherogenic lipids in combination with inhibitors of CaMKK2 (STO-609), cholesterol trafficking from the lysosome (U18666A), ER calcium release (Ryanodine), or ROS generation (butylated hydroxyanisole; BHA), and measured AMPK-specific signaling to ACC. Tunicamycin and A-769662 were used as positive controls for ER stress and AMPK activation independent of modified LDL treatment, respectively. AMPK signaling to ACC was stimulated in the presence of both oxLDL and agLDL. This was partially attenuated by the direct inhibition of CaMKK2 by STO-609, U18666A inhibition of cholesterol trafficking, and by inhibition of calcium release by Ryanodine (Figure 2C,D for agLDL and Supplementary Figure S2C,D for oxLDL). These effects were more pronounced in the agLDL-treated macrophages. Treatment with BHA did not alter AMPK signaling. Interestingly, while both oxLDL and agLDL treatments induced mild ER stress (increased expression of IREα), ER stress induction via tunicamycin did not result in augmented signaling to ACC. Importantly, AMPK activation in this experiment via A-769662 was used in the absence of ER stress-inducing agents.

**Figure 3:**
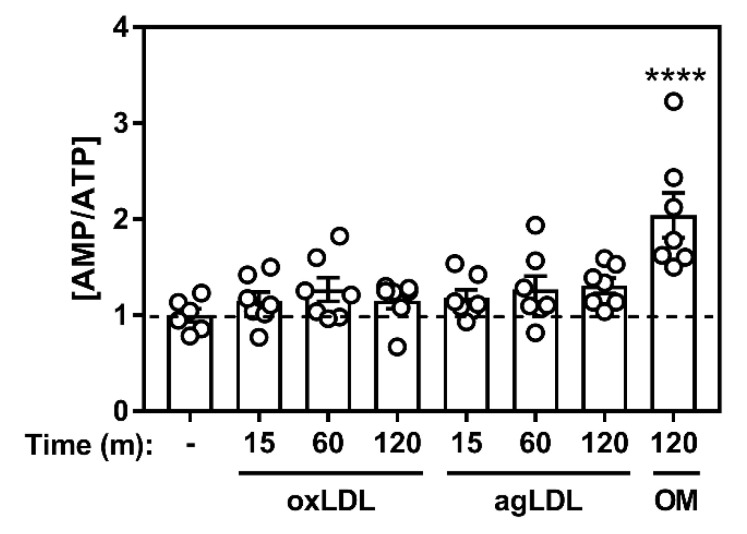
Acute incubation with atherogenic lipoproteins minimally shifts the energy status in cultured macrophages. Macrophages were cultured in the presence of oxidized (oxLDL) (50 µg/mL), agLDL (50 µg/mL), or oligomycin (OM) (5 µM) as a positive control. Data represent the mean ± SEM and expressed relative to naïve macrophage control (dashed line), where **** represents *p* < 0.0001 compared to vehicle-treated time 0 min.

LKB1 regulates AMPK activity in response to changes in adenine nucleotide ratios. We measured the levels of AMP and ATP in BMDM incubated with atherogenic lipids but observed no significant change in AMP:ATP ratio (Figure 3). Taken together, these results suggest that calcium/CaMKK2, but not ROS or energy nucleotides, play a role in mediating lipid-induced AMPK activation.

### 2.3. CD36 Links Atherogenic Lipids, AMPK Signaling, and Autophagy

**Figure 4:**
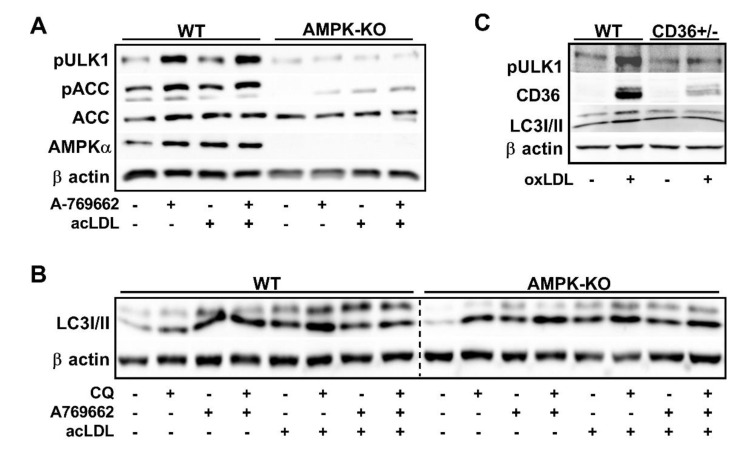
CD36 transmits lipid-induced AMPK activation signal in cultured macrophages. (**A**) Cells were treated with ± acLDL (50 µg/mL) and ± A-769662 (100 µM) for 24 h prior to harvest. (**B**) Macrophages were treated ± AMPK activation (A-769662; 100 µM), autophagy inhibition (chloroquine; 25 µM), and atherogenic lipoproteins (acLDL; 50 µg/mL). (**C**) WT and CD36-deficient macrophages were incubated with oxLDL for 24 h (50 µg/mL). pULK1 (150 kDa), pACC/ACC (260 kDa), pAMPKα/AMPKα (60 kDa), LC3II (14 kDa), CD36 (53 kDa), and β actin (37 kDa). (**A**,**C**) Total and phosphorylated proteins were determined from duplicate gels. Results are representative of three independent experiments from separate mice.

Autophagy is a highly conserved and regulated process that has been shown to be important for mobilizing cholesterol from macrophage foam cells [37]. There is evidence that upon lipid-loading of cultured macrophages, autophagy is initiated and bulk programs that control autophagy and lysosomal biogenesis are stimulated [34]. To link the observation that atherogenic lipids activated AMPK in macrophages, we treated WT and AMPKβ1-deficient macrophages (shown as AMPK KO in figures) with acLDL and assessed markers of autophagy. Importantly, as we have shown previously, AMPKβ1-null macrophages had a dramatic (~90%) reduction in AMPK signaling to ACC or ULK1, either in response to AMPK-specific activation (A-769662) or acLDL treatment (Figure 4A). To further investigate AMPK-dependent autophagy signaling, we treated WT and AMPKβ1-deficient macrophages with and without chloroquine (to inhibit autophagosome and lysosomal fusion). Compared to WT control cells, AMPK deficiency did not alter the potential autophagic flux as seen by the expression of LC3II relative to β actin in the presence of chloroquine. Interestingly, in response to acLDL-mediated lipid loading, LC3II is similarly augmented in both WT and AMPKβ1-null cells, however, an AMPK-dependent increase in LC3II content was observed with A-769662 (Figure 4B). Importantly, we were unable to compare between genotypes here as these results were from separate gels.

The scavenger receptor CD36 plays a pivotal role in the recognition and unregulated uptake of atherogenic lipids such as oxLDL [7]. There is also evidence that AMPK regulates CD36 and vice versa [38,39]. We next questioned whether CD36-mediated the activation of AMPK signaling in response to atherogenic lipids [39,40]. Treatment with oxLDL dramatically enhanced AMPK-specific ULK1 Ser555 phosphorylation and increased the conversion of LC3II in WT, but not CD36^+/−^ cells (Figure 4C), suggesting that CD36 plays a role in transmitting the atherogenic signal to AMPK, which in turn signals to regulate autophagy programs.

### 2.4. Macrophage AMPK Regulates Autophagy Signaling

**Figure 5:**
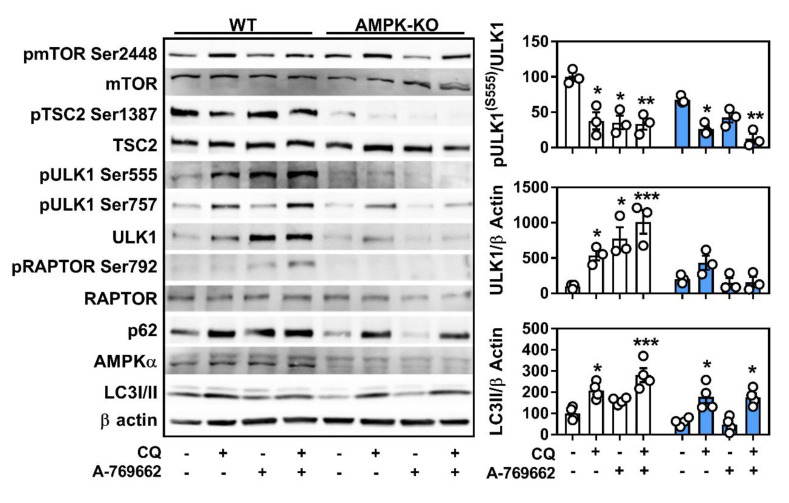
Macrophage AMPK signals through autophagy and is linked to autophagic protein expression. Signaling and flux through autophagy in response to AMPK expression and activation (A-769662 100 µM) and/or autophagy inhibition (chloroquine 50 µM) for 5 h. Total and phosphorylated proteins were determined from duplicate gels. Relative quantification for select targets is shown on the right. pmTOR/mTOR (280 kDa), pTSC2/TSC2 (200 kDa), pULK1/ULK1 (150 kDa), pRAPTOR/RAPTOR (150 kDa), p62 (62 kDa), AMPKα (60 kDa), LC3I/II (17/14 kDa), and β actin (37 kDa). Data are the mean ± SEM and is representative of at least three independent experiments from BMDM isolated from separate mice, where *, ** and *** represent *p* < 0.05, *p* < 0.01, and *p* < 0.001 compared to vehicle control for within genotype.

Independent of atherogenic lipid-loading, we were interested in how macrophage AMPK signals to regulate autophagy. As above, the lipidation of LC3I to LC3II and the protein content of p62 were increased in the presence of chloroquine in both WT and AMPKβ1-null cells (Figure 5). However, activation of AMPK did not reveal changes in autophagy flux that were AMPK-dependent. Therefore, we focused on upstream signaling pathways. In WT cells, AMPK activation increased the phosphorylation of ULK1 at Ser555 (an AMPK-specific site) without altering the levels of ULK1 Ser757 (an mTORC1-specific site) phosphorylation. Moreover, activation of AMPK was associated with slight increases in the phosphorylation of RAPTOR and TSC2, which are classically known to facilitate mTORC1 inhibition in WT but not AMPKβ1-null cells. However, in AMPKβ1-deficient macrophages, where mTORC1 inhibition of autophagy might be expected to prevail, ULK1 Ser757 phosphorylation was lower when compared to WT control cells. Interestingly, AMPK activation led to a potent upregulation of ULK1 protein content, whereas there was a dramatic reduction in ULK1 in AMPKβ1-null cells compared to the control. Chloroquine treatment also augmented ULK1 protein content, however, this was independent of AMPK signaling (Figure 5 and Supplementary Figure S3).

### 2.5. Transcriptional Control of Lysosomal Programs by Macrophage AMPK Is TFEB-Dependent

**Figure 6:**
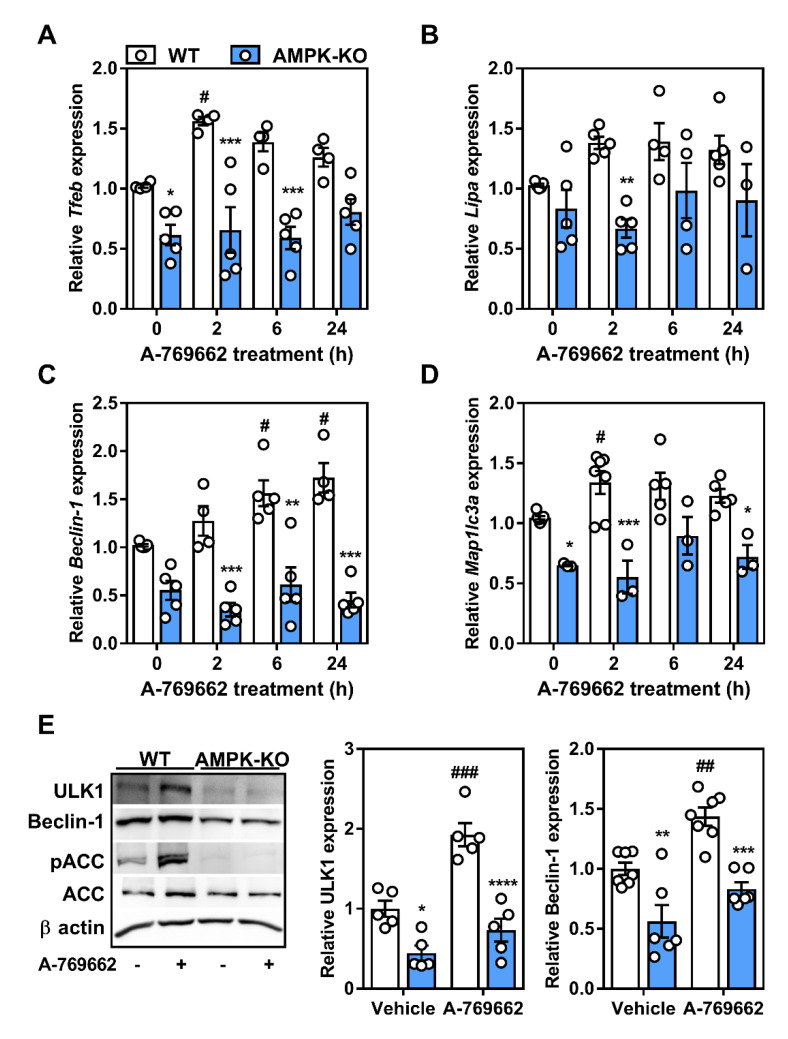
Macrophage AMPK activation leads to the upregulation of autophagy-related genes. Relative transcript expression in response to AMPK activation (A-769662 100 µM) over time for (**A**) Transcription factor EB (TFEB, encoded by *Tfeb*), (**B**) lysosomal acid lipase (*Lipa*), (**C**) Beclin-1 (*Beclin-1*), and (**D**) LC3 (*Map1lc3a*). Transcript expression was normalized to the average expression of *β-actin* and *Tbp.* (**E**) Protein expression of ULK1 and Beclin-1 were determined following treatment ± A-769662 (100 µM) for 24 h. ULK1 (150 kDa), Beclin-1 (52 kDa), pACC/ACC (260 kDa), and β actin (37 kDa). Data represent the mean ± SEM, where *, **, ***, and **** represent *p* < 0.05, *p* < 0.01, *p* < 0.001, and *p* < 0.0001 between genotypes, respectively, and where ^#^, ^##^, and ^###^ represents *p* < 0.05, *p* < 0.01, and *p* < 0.001 between treatments, as calculated by a 2way ANOVA.

It is known that AMPK signals ULK1 (at various sites) [27,41] to initiate autophagy. While we confirmed in macrophages that there was a downregulation of ULK1 expression in AMPK-deficient cells [24,29], we also observed a consistent upregulation of ULK1 protein in response to direct AMPK activation (Figure 5). Given the recent link between TFEB and AMPK, we queried whether acute activation of AMPK altered the transcript expression of several lysosomal and autophagic genes. Transcript expression of TFEB and regulated downstream targets Beclin-1, lysosomal acid lipase (LAL), and LC3 were all lower in AMPKβ1-deficient macrophages compared to the WT control cells (Figure 6A–D). Over the course of 24 h, the A-769662 WT-treated cells experienced a greater upregulation of these targets, whereas AMPKβ1-null cells remained mainly unchanged. We then confirmed that the protein expressions of TFEB-regulated ULK1 and Beclin-1 were significantly reduced in AMPKβ1-deficient BMDM compared to the WT control and that A-769662 increased the total protein expression of both ULK1 and Beclin-1 in macrophages from WT mice but not AMPKβ1-null cells (Figure 6E).

**Figure 7:**
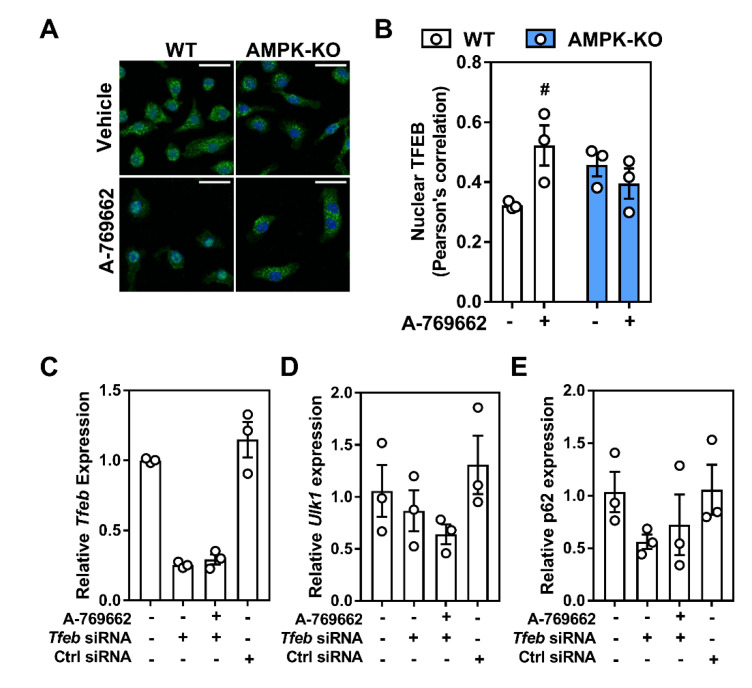
Macrophage AMPK activation regulates lysosomal-associated gene transcription through TFEB regulation. (**A**) Macrophages were treated for 2 h with A-769662 (100 µM). Shown is a representative immunofluorescent image z-projection for maximum intensity, scale bar is 25 µm (TFEB; Green, DAPI; Blue). (**B**) Pearson’s correlation coefficient for the co-localization of TFEB and DAPI across all z-stacks from (**A**), where 1 represents 100% co-localization and 0 represents 100% no co-localization. Macrophages were incubated with *Tfeb* or control siRNA followed by AMPK activation with 5 h incubation with A-769662 (100 µM). The transcript expressions of (**C**) *Tfeb*, (**D**) *Ulk1*, and (**E**) *Sqstm1* (gene encoding p62). Transcript expression was normalized to the average expression of *β-actin* and *Tbp.* Data represent the mean ± SEM, where # represents *p* < 0.05 between treatments, as calculated by a 2way ANOVA.

Given that TFEB is implicated in the regulation of these targets, we treated macrophages with A-769662 to activate AMPK and observed a dramatic shift in the nuclear enrichment of TFEB (Figure 7A,B). However, when TFEB mRNA levels were silenced, the transcript expression of downstream targets ULK1, Beclin-1, and p62 that were associated with A-769662-mediated AMPK activation remained unresponsive (Figure 7C–E). Taken together, this suggests that in macrophages, AMPK activation signals via TFEB to control lysosomal programs.

### 2.6. Lysosomal Programs Are Partly Regulated by Macrophage AMPK

**Figure 8:**
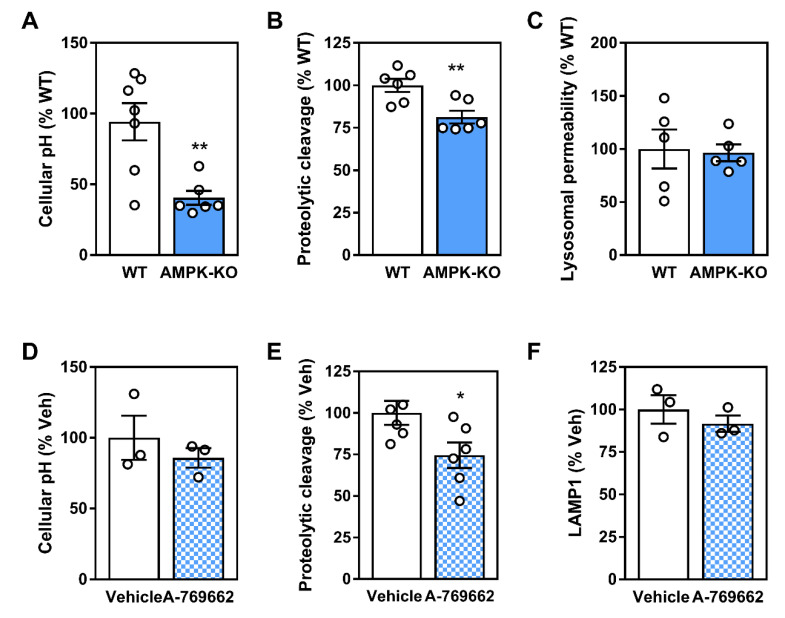
Macrophage AMPK expression and activation influence parameters for lysosomal homeostasis. WT and AMPKβ1-deficient macrophages were assessed for (**A**) cellular acidity, (**B**) DQ-ovalbumin proteolytic cleavage, and (**C**) lysosomal membrane permeability. WT and AMPKβ1-deficient macrophages were treated ± A-769662 (100 µM) for 24 h followed by measurement of (**D**) cellular acidity, (**E**) DQ-ovalbumin proteolytic cleavage, and (**F**) LAMP1 staining. Data represent the mean ± SEM, where * and ** represent *p* < 0.05 and *p* < 0.01 as calculated by the two-tailed Student’s *t*-test.

Since AMPK signaling plays a role in controlling transcript levels of important autophagy and lysosomal components, we next determined the functional parameters related to lysosomal function in BMDM from WT and AMPKβ1-deficient mice. While lysosomal homeostasis is a highly dynamic process, AMPK deficiency was associated with a significant reduction in the overall number of acidic compartments (taken to broadly represent lysosomal and endosomal compartments) while trending toward a lower ability to cleave a set substrate (interpreted as proteolytic cleavage) compared to WT control cells (Figure 8A,B). There were no differences in measures of lysosomal membrane permeability (Figure 8C). While representing static measures, these results support a broad role for AMPK signaling in facilitating lysosomal programs. Finally, we assessed if these functional measures of lysosomal homeostasis were augmented with AMPK activation. Surprisingly, AMPK activation via A-769662 had no effect on total cellular acidity or total LAMP1 staining and had a suppressive effect on proteolytic cleavage (Figure 8D–F).

### 2.7. Atherogenic Lipids Overwhelm AMPK Regulation of Lysosomal Programs

**Figure 9:**
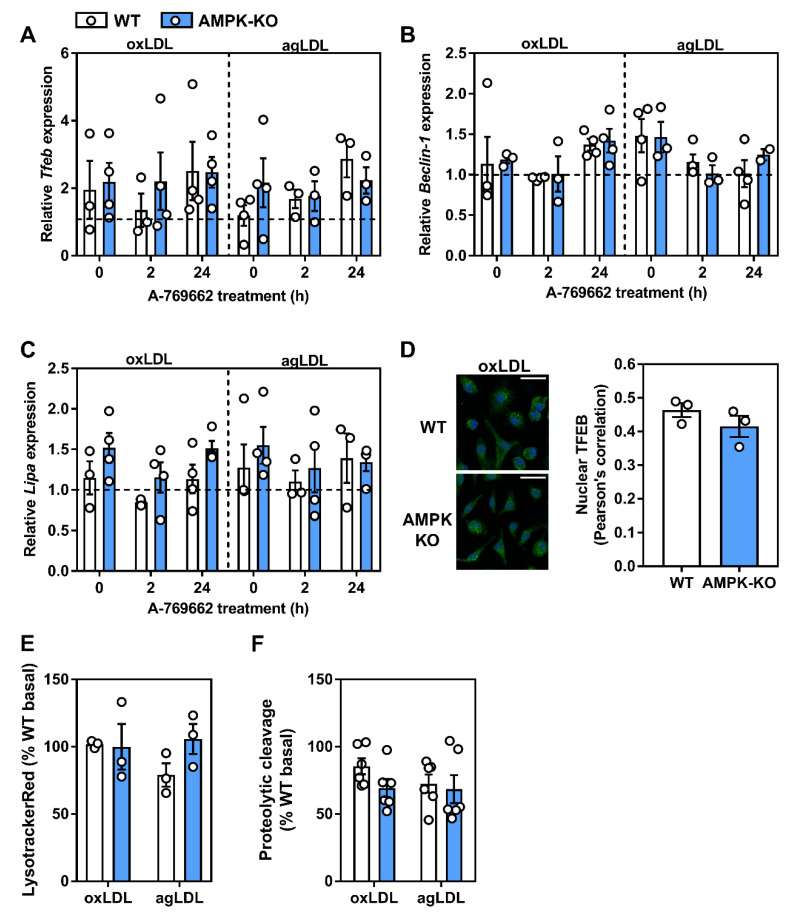
Atherogenic lipoproteins uncouple AMPK from its regulation of lysosomal-associated gene transcription. WT and AMPKβ1-deficient macrophages were cultured for 48 h in ox-LDL- or agLDL-containing media (50 μg/mL each), followed by a 24 h-treatment with A-769662 (100 μM). Relative transcript expression of (**A**) *Tfeb*, (**B**) *Beclin-1* and (**C**) *Lipa* (lysosomal acid lipase) was determined by normalizing to the average of *β actin* and *Tbp*. The horizontal hashed line represents the expression of untreated WT BMDM. (**D**) WT and AMPKβ1 knockout cells were treated with oxLDL (2 h; 50 µg/mL) and imaged. Representative immunofluorescent image z-projection for maximum intensity, scale bar was 25 µm (TFEB; Green, DAPI; Blue). Pearson’s correlation coefficient quantification of the entire z-stack (right). WT and AMPKβ1-deficient cells were co-cultured with oxLDL and agLDL (50 µg/mL) for 24 h followed by determination of (**E**) Lysotracker Red staining and (**F**) DQ-ovalbumin proteolytic cleavage. Data represent the mean ± SEM.

It has been shown that atherogenic lipids acutely stimulate a transcriptional lysosomal program [34]. Given our data that atherogenic lipids activate AMPK and that AMPK activation can influence TFEB-regulated lysosomal programs, we hypothesized that AMPK is responsible for the lipid-induced stimulation of lysosomal pathways. Using BMDM, we incubated WT and AMPKβ1-null cells with atherogenic lipoproteins for 3–12 h and assessed the transcript expression of TFEB and two downstream effector genes. Unexpectedly, we observed no induction of TFEB, Beclin-1, or LAL transcript by either oxLDL or agLDL (Supplementary Figure S4). Moreover, in oxLDL- or agLDL-derived foam cells, A-769662 had no effect on TFEB-related transcript levels (Figure 9A–C). Complementary to this, upon oxLDL treatment, no differences were observed in the amount of TFEB in the nucleus. Finally, when cells were primed with atherogenic lipids, AMPK signaling and regulation of lysosomal function was largely uncoupled (Figure 9E,F).

## 3. Discussion

The unregulated uptake and retention of cholesterol-rich lipoproteins is a hallmark of atherogenesis [10,42,43], yet a complete understanding of the molecular mechanisms that regulate these processes is still lacking. In this study, we aimed to determine if AMPK signaling was affected by treatment with atherogenic lipoproteins and if so, the potential mechanism(s) and consequences. Using bone marrow macrophages, we demonstrated that AMPK signaling was stimulated in response to various types of atherogenic lipoproteins via the calcium activated CaMKK2. However, although AMPK is important in macrophages for the induction and maintenance of lysosomal and autophagic pathways (TFEB), treatment with atherogenic lipoproteins negates these effects.

AMPK signaling is required for proper monocyte-macrophage differentiation and in the human THP-1 monocyte cell line, as exposed to differentiating stimuli (including oxLDL, phorbol esters, and 7-ketocholetsterol), resulting in AMPK activation [24]. To our knowledge, our studies are the first to investigate AMPK signaling in response to model (acLDL) and physiological (oxLDL/agLDL) atherogenic stimuli in BMDM used at concentrations in keeping with the literature. We first focused on a potential mechanism to explain how exposure to these modified lipoproteins could lead to AMPK activation, which can occur in response to glucose deprivation (lysosomal activation), adenine nucleotide fluctuations (AMP/ADP binding to the γ subunit of AMPK), intracellular calcium release (via CaMKK2), and most recently, binding of long chain acyl-CoA [44]. Since it has been shown that oxLDL is capable of triggering ER stress, a process linked to alterations in cytosolic calcium flux [3,35,36], we reasoned that calcium-stimulated CaMKK2 may be responsible. Using tunicamycin and A-769662 as positive controls for ER stress and AMPK activation, respectively, oxLDL and agLDL treatments caused a mild ER stress response. Since tunicamycin-induced ER stress did not affect AMPK signaling, it would be misleading to conclude that ER stress itself is responsible; however, in lipid-loaded macrophages, it appears that AMPK signaling is largely affected by the release of calcium and the upstream activation of CaMKK2.

Energy fluctuations are reflected by changes in the AMP:ADP:ATP ratios. Upon treatment with atherogenic lipoproteins, there was a small trend toward an increase in the AMP:ATP ratios (Figure 3). The activity of the upstream kinase LKB1 has been described as constitutively active, making its phosphorylation and activation of AMPK a function of nucleotide binding and AMPK conformation [15]. Our results do not rule out the potential for LKB1-mediated AMPK activation, which may be responsive to changes in energy metabolism upon lipid loading [45]. Interestingly, binding of long chain acyl-CoA molecules to a conserved region of the AMPK β1 subunit causes allosteric activation (providing endogenous allosteric activation). Therefore, it remains entirely plausible that fatty acids from modified LDL metabolized in a manner that facilitated the allosteric activation of AMPK. Finally, AMPK activation in response to glucose deprivation has been shown to be intricately tied to glycolytic flux (fructose 1,6 bisphosphate binding to aldolase being the key step) and the lysosomal localization of the AMPK-LKB1-Axin activating complex [46]. The physiological relevance of this activation pathway is still being evaluated and provides the basis for exploring whether plaque-resident cells have sufficient access to glucose during atherosclerosis. However, in our study, BMDM were cultured in a surplus of glucose in the media (25 mM), making this an unlikely contributing mechanism for AMPK activation.

Macrophages undergo a classic upregulation of scavenger receptors upon differentiation from monocytes, which in turn facilitate the unregulated uptake of modified lipoproteins. CD36 is an important receptor that has been implicated in hepatic AMPK signaling [39]. We wondered if in cultured macrophages whether CD36 transmitted the lipid-associated signal that caused AMPK activation. We observed that CD36 deficiency dramatically reduced the phosphorylation of ULK1 and limited the levels of LC3-II, supporting that CD36 expression controls downstream signals to AMPK and subsequently, autophagy.

Our results indicate that autophagy is active in macrophages even in the absence of AMPK signaling. However, this is where complexities in autophagic flux interpretation come into play. While we can conclude that autophagic flux is maintained in both WT and AMPKβ1-deficient macrophages, AMPK activation resulted in more LC3 lipidation in WT cells, such that when autophagy was inhibited with chloroquine, there were no observable differences in basal or lipid-loaded conditions (Figure 4B). Moreover, chloroquine treatment in AMPKβ1-null macrophages showed a consistent increase in p62 protein content that was basally lower compared to the WT control cells and not upregulated in response to AMPK activation (Figure 5). Therefore, our data showed that AMPK signaling increases the expression of autophagic machinery and potentially its capacity, but the limitations with measuring bulk autophagic flux by immunoblotting methods prevent further interpretation [47]. Future work is warranted using more autophagy-specific tools [48].

Our understanding of the role of AMPK-specific signaling in autophagy is continually expanding. While most studies have focused on acute, phospho-regulation of downstream targets, a clearer picture of transcriptional regulation is beginning to emerge. TFEB, a master regulator of lysosomal and autophagic programs, has recently been shown to be regulated both directly and indirectly by AMPK [29,32,33]. Our data suggest that normal AMPK signaling is required in murine macrophages to maintain regular transcriptional and cellular control over lysosomal programs. Since the discovery of AMPK signaling to ULK1, there has been little focus on the potential transcriptional control of ULK1 in response to AMPK signaling. Our data corroborate recent work demonstrating that in myeloid cells, levels of ULK1 and Beclin-1 are drastically diminished when AMPK expression is restricted [24,49]. However, we show for the first time in macrophages that AMPK activation via A-769662 results in an upregulation of ULK1 and Beclin-1 protein levels. This was associated with increased TFEB-regulated gene expression as well as increased TFEB nuclear localization. Furthermore, given that atherogenic lipoproteins activated AMPK, which in turn can influence TFEB-regulated pathways, it is possible that AMPK mediates part of the compensatory induction of lysosomal biogenesis by atherogenic lipids reported previously by others [34].

We predicted that atherogenic lipoproteins would regulate transcriptional expression of lysosomal-associated genes through AMPK activation. However, our results were complicated by the fact that neither agLDL nor oxLDL had any effect on TFEB-associated transcript expression in WT macrophages, which had been previously shown [34]. Additionally, and in contrast to basal treatment without lipids, the expression levels in AMPKβ1-deficient cells treated with oxLDL were at times opposite. While there could be batch-to-batch and technical/experimental variation in preparing oxLDL (also in-house preparation vs. commercial suppliers), the type of macrophage presents the biggest discrepancy between ours and the published results [34]. Thioglycolate-elicited peritoneal macrophages are a heterogeneous population of infiltrating monocyte-derived macrophages, which are often considered more activated in comparison to the homogenous population of bone marrow-derived cells [50]. The metabolic and inflammatory characteristics of these two primary murine macrophages have been investigated and could provide an explanation as to the discrepancy. Future work using elicited peritoneal and bone marrow-derived cells from WT and AMPKβ1-null mice would help bridge the divide between results.

Our data support that physiological, modified, and atherogenic lipoproteins activate macrophage AMPK signaling via changes in calcium homeostasis. The activation of macrophage AMPK resulted in an upregulation of lysosomal and autophagic pathways, responses that were blunted or not observed in AMPKβ1-deficient macrophages. Although endogenous AMPK signaling was important for various aspects of lysosomal homeostasis, in the presence of atherogenic lipoproteins, AMPK activation was insufficient to augment the same lysosomal programs. In the context of progressing atherosclerosis, there could be a futile cycle, whereby plaque-resident macrophages are continually exposed to atherogenic stimuli that activate AMPK/TFEB related gene programs. However, the potential benefit stemming from acute AMPK activation including the stimulation of autophagy and lysosomal pathways are paradoxically not engaged. Rather, autophagy becomes progressively defective during the progression of atherosclerosis despite potential alterations in AMPK signaling [51]. Endogenous regulation of AMPK by pro-atherogenic lipoproteins does not rescue defective autophagy in cultured atherosclerotic macrophages, however, whether pharmacological activators of AMPK are sufficient to overcome this hurdle remains to be tested.

## 4. Materials and Methods

**Animals:** The generation and characterization of the AMPKβ1 knockout mice have been previously described [52]. Mice were generated from heterozygous pairings so that control mice were littermates. Mice were maintained on a 12-h light/dark cycle (lights on at 7:00 a.m.) and housed at 23 °C with bedding enrichment in a ventilated cage system. Male and female mice aged 8–16 weeks were used for the generation of primary macrophages as described below. CD36-deficient cells were isolated from the femurs of CD36 heterozygous mice, a kind gift from Dr. Kathryn Moore at NYU. All animal procedures were performed at the University of Ottawa and approved by the University of Ottawa Animal Care Committee (Protocol BMI-1863, Approval date: 3 September 2014).

**Isolation and culturing BMDM:** Mice were anesthetized with ketamine (150 mg/kg) and xylazine (10 mg/kg), then euthanized by cervical dislocation. Each hind leg was carefully excised from the mouse, then the legs were cleaned of soft tissue until bones were completely devoid of all tissue. Both ends of the femur and tibia were carefully cut, bones were then placed a 0.5 mL tube containing a hole punctured at the bottom with 18-gauge needle at the bottom prior. The 0.5 mL tube containing bones was placed in a sterile 1.5 mL microfuge tube with 100 µL of culture media; Dulbecco’s modified Eagle’s medium (DMEM) supplemented with 10% fetal bovine serum and 100 U/mL penicillin-streptomycin (culture media). The tubes were centrifuged on a benchtop microcentrifuge at 4000 rpm for 5 min. The bones were discarded and 900 µL of culture media was added to each tube containing the bone marrow pellets, the pellets were gently re-suspended with gentle pipetting, and then strained through a 40 µm cell strainer into culture media. Bone-marrow containing media was supplemented with 15% L929-conditioned media (and additional culture media) and plated within 150 mm tissue culture plates as needed. Cells were placed in a humidified incubator (37 °C with 5% CO_2_) for 7–8 days to allow for macrophage differentiation. Following this, media were removed from each 150 mm plate, cells were washed once with PBS, and then gently scraped in culture media. Cells were counted and seeded as necessary to meet the experimental work.

**Protein signaling experiments:** BMDM was harvested and plated within a 6-well tissue culture plate at a density of 1.2 million cells per well (duplicate wells plated for each experimental condition) in culture media. All experiments began with removal of culture media, cells were washed once with ice-cold PBS. Following, the PBS was removed and 1 mL of fresh culture media was added to each well with experimental treatments. Experiments were conducted so that the longest time-points (if applicable) were treated first to ensure all conditions were finished simultaneously. At the experimental endpoint, all media was removed and cells were washed with PBS. PBS was removed and cells were scraped in 80 µL lysis buffer (50 mM Tris.HCl pH 7.5, 150 mM NaCl, 1 mM EDTA, 0.5% Triton X-100, 0.5% NP-40, 100 µM Na_3_VO_4_), supplemented with a protease inhibitor cocktail (PIC) tablet. Duplicate lysates were combined and placed in a new 1.5 mL microfuge tube and snap frozen with liquid nitrogen. Lysates were thawed on ice, following samples were centrifugated at 14000 RPM at 4 °C for 5 m. The pellet was discarded and the lysate was collected and placed in a new 1.5 mL tube and stored at −80 °C.

**Protein quantification, normalization, and preparation:** Total protein quantification was determined using the Pierce™ BCA Protein Assay Kit as per the manufacturer’s instructions under the microplate procedure and reading samples with Biotek’s Gen5 software. Following quantification, protein samples were normalized to the 1.34 µg/µL in lysis buffer, then an aliquot was further diluted in 4X loading dye; 200 mM Tris-HCl pH 6.8, 400 mM dithiothreitol (DTT), 8% sodium dodecyl sulfate, 0.4% bromophenol blue, and 40% glycerol. Protein samples in loading dye were then boiled at 95 °C for 5 min. Samples were stored at −20 °C until ready for analysis.

**LDL aggregation:** 1 mL of 5 mg/mL LDL was vortexed at max speed for precisely 60 s.

**Immunoblotting:** Equalized protein samples containing loading dye were loaded (15 µg protein/well) and electrophoresed onto either a 4–20% or 8% SDS-PAGE gel (110 volts for ~1.75 h). For analysis of the relative phosphorylation of target proteins, duplicate gels were electrophoresed in tandem to probe for phosphorylated and total levels for target proteins. Following electrophoresis, each gel was carefully removed and transferred onto PVDF membranes using Bio-Rad’s Trans-Blot Turbo system (25 V, 2.5 amps, for 18 min). Membranes were then blocked in TBST supplemented with 5% *w/v* BSA with gentle rocking at room temperature for 1 h. Membranes were then cut at appropriate molecular weights to isolate multiple proteins of interest per membrane. Each membrane fraction was incubated in primary antibody solution (TBST supplemented with 5% BSA and 1:1000 dilution of primary antibody of interest) overnight at 4 °C. The following day, the primary antibody solution was collected and membranes were washed four sequential times in TBST for 5 min with gentle rocking at room temperature. After washing, membranes were incubated with anti-rabbit HRP conjugated secondary antibody (TBST supplemented with 5% BSA and 1:10,000 dilution of anti-rabbit secondary) for 1 h at room temperature with gentle rocking. Following this, the secondary-antibody solution was discarded and membranes were washed four times as previously done. Image acquisition was completed by gently drying membranes then briefly incubating in the Clarity ECL substrate mix. Luminescence was measured using the General Electric’s ImageQuant LAS4000 system, while obtained image files were processed and quantified using ImageJ analysis software.

**Relative transcript expression experiment:** BMDM was harvested and plated within a 12-well tissue culture plate at a density of 0.6 million cells per well (triplicate wells plated for each experimental condition) in culture media. All experiments began with removal of culture media, cells were washed once with ice-cold PBS. Following this, the PBS was removed and 0.5 mL of fresh culture media was added to each well with experimental treatments. Experiments were conducted so that the longest time-points (if applicable) were treated first to ensure all conditions were finished simultaneously. At the experimental endpoint, all media were removed and 0.5 mL of TriPure reagent was added directly to the cells. TriPure containing plates were flash frozen by incubating the plate in a Styrofoam container with a shallow level of liquid nitrogen. Plates were thawed on ice, then samples in TriPure were collected following visual confirmation that adherent cells were not present (ensuring proper lysis) and placed within a sterile 1.5 mL microfuge tube (replicates were collected separately). Samples were then either stored at −80 °C or subjected to RNA isolation (explained below). For siRNA experiments, Silencer Select siRNA against mouse *Tfeb* (s74860 and s74861) as well as a negative control siRNA (4390843) (25 pmol) (Thermo Fisher Scientific, Mississauga, ON, Canada) were transfected into BMDM for 72 h with Lipofectamine RNAiMAX, as per the manufacturer’s instructions. For *Tfeb* knockdown, s74860 targeted exon 1 while s74861 targeted the junction between exon 6 and 7. While both were tested, only the results from s74861 are shown.

**RNA Isolation:** RNA isolations were performed using Roche’s TriPure Reagent Kit as per the manufacturer’s instructions. Following isolation, the RNA pellet was then resuspended in 30 µL of nuclease free water (Wisent, Saint-Jean-Baptiste, QC, Canada) and incubated at 58 °C for 15 m. Samples were stored at −80 °C until quantification and synthesis of cDNA were ready to be performed.

**RNA quantification:** RNA concentration and purity were assessed using a take-3 plate reader. In brief, 2 µL of sample was placed on a corresponding location within the take-3 plate in duplicate using nuclease free water as a blank. Samples were then measured using Gen5 software, samples with a 260/280 absorbance ratio less than 1.8 or greater than 2.0 were removed from assessment. All samples were then normalized to 20 ng/mL in nuclease free water, now ready for cDNA synthesis.

**cDNA synthesis:** Genomic DNA was removed from all RNA samples using an AccuRT Genomic DNA Removal Kit as per the manufacturer’s instructions. Following this, cDNA synthesis from RNA was performed using Applied Biological Material’s 5X All-In-One RT Master mix product as per the manufacturer’s instructions. Following cDNA synthesis, samples were further diluted 1:20 with nuclease-free water prior to transcript expression analysis.

**Quantitative PCR:** Quantitative PCR reactions were performed within 0.1 mL 4-Strip tubes using 4.75 µL of diluted cDNA template, 0.25 µL of the TaqMan primer/probe set of interest, and 5 µL of PCR master mix and run on a Rotorgene Q in a two-step amplification reaction.

**Immunofluorescent labeling:** Immunofluorescent experiments were conducted on 8-well chamber slides where each chamber was seeded with 5 × 10^4^ BMDM. Upon experimental completion, all media were removed and each chamber slide was washed once with PBS. PBS was removed, and cells were fixed for 20 min with 300 µL of 2% PFA at room temperature. The fixative was removed and chambers were washed twice with PBS. PBS was removed and fixed cells were blocked/permeabilized for 1 h at room temperature with PBS supplemented with 300 µL PBS supplemented with 5% fatty-acid free BSA, 0.2% Triton X-100, and 0.1% Tween-20. The blocking/permeabilizing buffer was removed, chambers were washed once with PBS, then 300 µL of anti-TFEB antibody solution (PBS supplemented with 1:100 anti-TFEB primary antibody, 2% fatty acid-free BSA, 0.2% Triton X-100, 0.1% Tween-20) was added. All chamber slides were incubated in primary antibody solution overnight at 4 °C. The following day, the antibody solution was removed, and the chambers were washed twice with PBS. PBS was removed and chamber slides were incubated in 300 µL secondary antibody solution (PBS supplemented with 1:100 anti-mouse Alexa 488-conjugated secondary antibody, 2% fatty acid-free BSA, 0.2% Triton X-100, 0.1% Tween-20) for 1 h at room temperature shielded from light. Following this, the secondary antibodies solution was removed and chambers were washed once with PBS. PBS was removed and 300 µL of 300 nM DAPI made in PBS was added to each chamber and left at incubation temperature for 5 m shielded from light. Following this, the DAPI solution was removed and all chambers were washed three times with PBS. Chambers were stored in 300 µL PBS at 4 °C shielded from light until image acquisition was ready to be performed.

**Immunofluorescent imaging and nuclear colocalization:** Following immunolabeling, multiple Z-stack images spanning the entirety of the cell at 50 µm increments were taken at 20X using a Zeiss LSM800 AxioObserver Z1 confocal microscope. Image files were processed using ImageJ software and representative Z-projection for maximum intensities were used for representative images. Co-localization between DAPI and TFEB channels throughout all z-stacks (23 slices) was performed using the EZColocalization plugin in ImageJ software as reported [53].

**AMP and ATP determination:** BMDM was harvested and plated within a 100 mm tissue culture plate (Thermo Fisher Scientific, Mississauga, ON, Canada) at a density of 5 million cells per plate in culture media (duplicate plates were seeded for each experimental condition). At the experimental endpoint, media were removed from each plate and cells were washed once with ice-cold PBS. Following this, 250 µL ice-cold perchloric acid (10% *v*/*v* HClO_4_, 25 mM EDTA) was added and cells were scraped/lysed with a rubber policeman. Cellular extracts were transferred to a sterile 1.5 mL microfuge tube and vortexed briefly. Samples were left to incubate on ice for 30 min, then precipitated proteins were spun down at 8000× *g* for 2 min at 4 °C. A 200 µL aliquot was taken and placed in a new microfuge tube, and the samples’ pH was adjusted to 6.5–7 using ~130 µL KOH/MOPS (2 N KOH/0.3 M 3-N-morpholino propane sulfonic acid) with careful mixing and confirmation using pH strips. Lysate was clarified by centrifugation at 8000× *g* for 2 min at 4 °C. A 200 µL aliquot of neutralized supernatant fraction was pipetted into a new 1.5 mL microfuge tube and stored at −80 °C until ready for high performance liquid chromatography measurement (HPLC). HPLC measurements were performed as described [54].

**Reagents and antibodies:** 4′,6-Diamidino-2-Phenylindole, 2-(4-amidinophenyl)-1H-indole-6-carboxamidine (DAPI; D1306), BCA™ Protein Assay (PI-23225), Dextran, Tetramethylrhodamine, 10,000 MW, Lysine Fixable (D1817), DFQ™ Ovalbumin (D12053), DMSO (23730571), Fatty Acid-Free Powder (BP9704100), Hydrochloric Acid (HCl) (8732113), Isopropanol (A416-4), Lipofectamine™ RNAiMAX Transfection Reagent (13778075), ProLong™ Gold Antifade Mountant (P36930), Penicillin/Streptomycin (SV30010) and Tween-20 (BP377-500) were from Thermo Fisher Scientific, Mississauga, ON, Canada. 5X All-In-One RT MasterMix with AccuRT Genomic DNA Removal Kit (G492) was from Applied Biological Material, Richmond, BC, Canada. A-769662 (844499-71-4) was from AdooQ Bioscience, Irvine, CA, USA. LDL (J65039), oxidized LDL (J65591) and acetylated LDL (J65029) were from Alfa Aesar, Tewksbury, MA, USA. Albumin (Albumin, Bovine, Serum, Heat Shock Isolation, Fraction V. Min. 98%; ALB001.250), Chloroform, ACS, Reagent Grade, 1 L (CCL402.1), Dithiothreitol (DTT), Electrophoresis Grade (DTT001.10), Potassium Hydroxide, Reagent Grade (PHY202.500), NP-40 (NON999.500), Paraformaldehyde (PAR070.500), Sodium Chloride (SOD001.1), Sodium Orthovanadate (SOV664.10), Triton X-100 (TRX777.500) were purchased from BioShop Canada Inc., Burlington, ON, Canada. Butylated hydroxyanisole (B1235-5G), Chloroquine (C6628-25G), MOPS (M3183-25G), U18666A (U3633), Perchloric Acid ACS reagent 70% (311421-250mL), Tunicamycin (T7765), Complete Protease Inhibitor Cocktail Tablets Mini, EDTA-Free-30 units (4693159001) were from Sigma-Aldrich, Oakville, ON, Canada. Clarity Western ECL Substrate (170-5060) was from BioRad, Mississauga, ON, Canada. CytoPainter Lysosomal Staining Kit-Blue Fluorescence (ab112135) and STO-609-acetic acid (ab141591) were from Abcam, Burlingame, CA, USA. DMEM (319-005-CL), EDTA (625-060-CG), Fetal bovine serum (095-150), PBS (311-010-CL), Water Rnase and Dnase free (809-115-CL) was from Wisent, Saint-Jean-Baptiste, QC, Canada. Ethanol (P016EAAN) was from Commercial Alcohols, Toronto, ON, Canada. Anti-Mouse CD107a (LAMP-1) eFluor 450 (48-1071-82), Rat IgG2a Isotype Control eFluor 450 (48-4321-80), Oligomycin A (4110 n/5) and Ryanodine (13-291) were from Cedarlane, Burlington, ON, Canada. Opti-MEM Reduced Serum Medium (31985-070) was from Invitrogen, Carlsbad, CA, USA. TriPure (11667165001) was from Roche, Laval, QC, Canada. Tris (TB0196) was from Bio-Basics Inc., Markham, ON, Canada. Acetyl-CoA Carboxylase (3676), AMPKα (5831), Anti-rabbit IgG HRP-linked Antibody (7074S), ATF-6 (65880), Beclin-1 (3495), β-Actin (5125), CHOP (2895), LC3A/B (D3U4C) (12741), mTOR (2983), Phospho-ACC Ser79 (11818), Phospho-AMPKα Thr172 (2535), Phospho-mTOR Ser2481 (2974), Phospho-Raptor Ser792 (2083), Phospho-Tuberin/TSC2 Ser1387 (23402), Phospho-ULK1 Ser555 (5869), Phospho-ULK1 Ser757 (6888), Raptor (2280), SQSTM1/p62 (5114), Tuberin/TSC2 (D93F12) (4308), ULK1 (8054) were from Cell Signaling Technologies, Danvers, MA, USA. TFEB, Monoclonal Antibody (MBS120432) was from Mybiosource, Inc., San Diego, CA, USA.

**Statistical analyses**: All statistical analyses were completed using GraphPad Prism 7.03 (GraphPad Software Inc., San Diego, CA, USA). Comparison between two groups was made using an unpaired, two-tailed Student’s *t*-test. Comparisons between more than two treatment groups were made using a one-way ANOVA analysis with a Dunnett’s test for multiple comparisons. For experiments comprising more than two groups (genotype and treatment), a two-way ANOVA was used with a Sidak or Holm–Sidak test for multiple comparisons. Significant differences are described in the figure legends. All data were expressed as mean ± SEM, unless specified in the figure legend.

## Supplementary Materials

Supplementary materials can be found at https://www.mdpi.com/1422-0067/21/23/9033/s1.

## Abbreviations

acLDL	acetylated-LDL
ACC	acetyl-CoA carboxylase
agLDL	aggregated-LDL
AMPK	AMP-activated protein kinase
BMDM	bone marrow-derived macrophages
CaMKK2	calmodulin-dependent protein kinase kinase-2
LAL	lysosomal acid lipase
LDL	low-density lipoprotein
LKB1	liver kinase B1
mTORC1	mechanistic target of rapamycin complex 1
oxLDL	oxidized-LDL
TFEB	transcription factor EB
