# Supplementary material for: Foam Cell Induction Activates AMPK But Uncouples Its Regulation of Autophagy and Lysosomal Homeostasis
Source: Int J Mol Sci. 2020 Nov 27;21(23):9033. doi: 10.3390/ijms21239033 (PMC7730064; doi:10.3390/ijms21239033)
Supplement: Supplementary file 1 [file ijms-21-09033-s001.pdf]

Supplementary Figures

Foam cell induction activates AMPK but uncouples its regulation of autophagy and lysosomal homeostasis

Nicholas D. LeBlond[1,2,3], Julia R. C. Nunes[1,2,3], Tyler K.T. Smith[1,2,3], Conor O'Dwyer[1,2,3], Sabrina Robichaud[1,4], Suresh Gadde[1,2,5], Marceline Cote[1,2,3], Bruce Kemp[6,7], Mireille Ouimet[1,4] and Morgan D. Fullerton[1,2,3]*

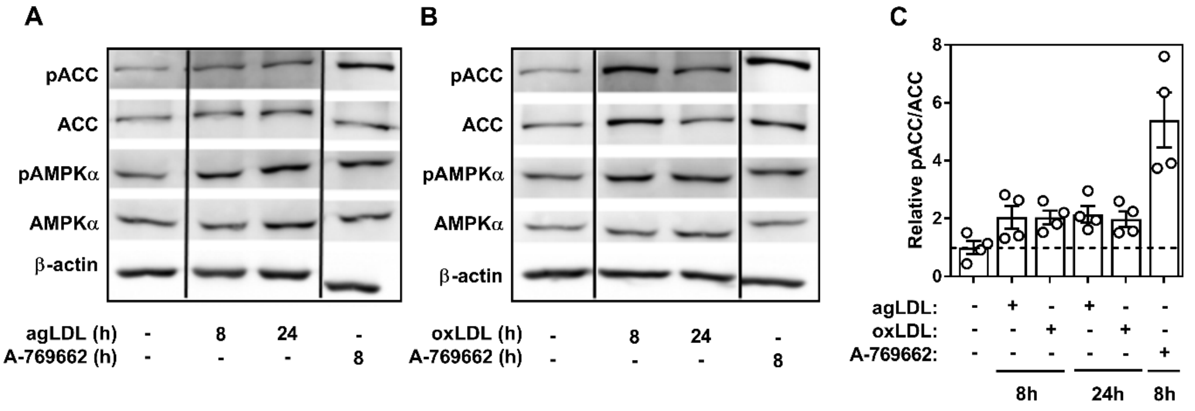

Supplementary Figure S1. Atherogenic lipoproteins agLDL and oxLDL enhance AMPK-specific signaling to ACC. (A) Representative immunoblot depicting increased AMPK signaling in response to incubation with agLDL (50 µg/mL) for 8 and 24 h. A-769662 (8h; 100 µM) was used as a positive control for AMPK activation. (B) Representative immunoblot depicting increased AMPK signaling in response to incubation with oxLDL (50 µg/mL) for 8 and 24 h. A-769662 (8h; 100 µM) was used as a positive control for AMPK activation. (C) Quantification of relative signaling to ACC at Ser79 in response to 8 and 24 h incubation with atherogenic lipoproteins agLDL and oxLDL. pACC and ACC (260 kDa), pAMPKα and AMPKα (60 kDa) and β actin (37 kDa). Data represent the mean ± SEM.

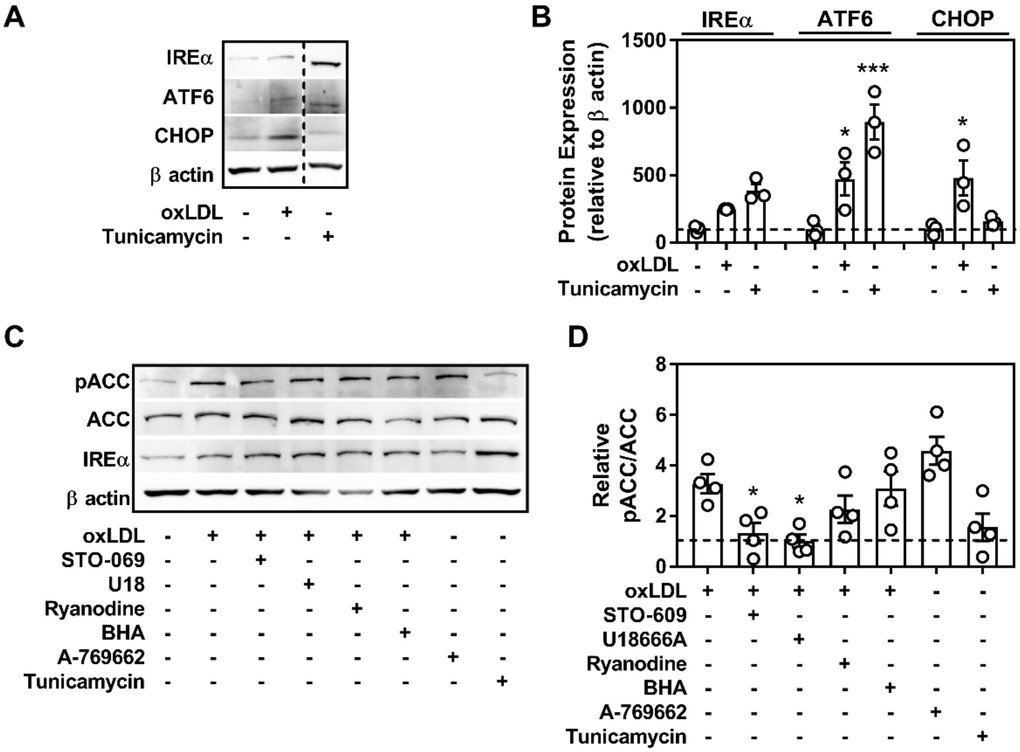

Supplementary Figure S2. oxLDL induces ER stress and activates AMPK partially through CaMKK2 signaling in cultured macrophages. (A) Representative immunoblots depicting the induction of ER stress by up-regulation of its markers IREα (130 kDa), ATF6 (90 kDa) and CHOP (27 kDa), in response to agLDL (50 μg/mL). (B) Relative quantification of ER stress markers relative to β actin. (C) Assessing the potential role that CaMKK2 (STO-609; 25 μM), cholesterol trafficking (U18666A; 1 μM), ER-calcium release (Ryanodine; 1 μM), and ROS (BHA; 100 μM) play in activating macrophage AMPK in response to agLDL (50 μg/mL) (D) Relative quantification of pACC to ACC signal, shown relative to vehicle control treated (no oxLDL) cells (hashed line). Macrophages isolated from WT mice were incubated with agLDL (50 μg/mL) along with inhibitors for 18 h. A-769662 (100 μM) and Tunicamycin (2.5 μg/mL) were used as a positive control for AMPK activation and ER stress, respectively. Data is mean ± SEM and is representative of at least 3 independent experiments using BMDM isolated from separate mice, where

* and *** represent p<0.05 and p<0.001 compared to vehicle control for (B) and compared to oxLDL-treated cells in (D).

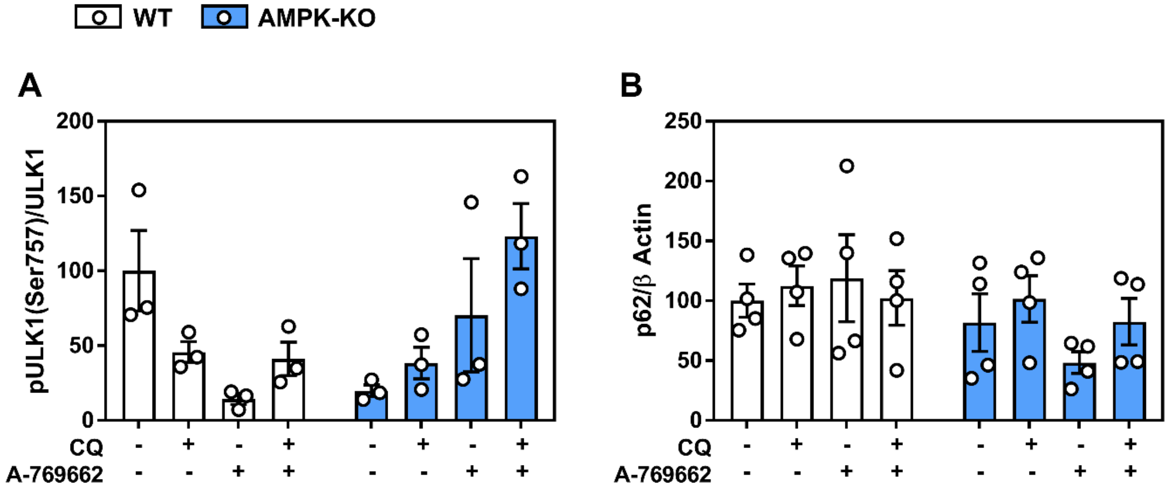

Supplementary Figure S3. Macrophage AMPK signals through autophagy and is linked to autophagic protein expression. Relative quantification of indicated protein ratios. Data is mean±SEM and is representative of at least 3 independent experiments from BMDM isolated from separate mice.

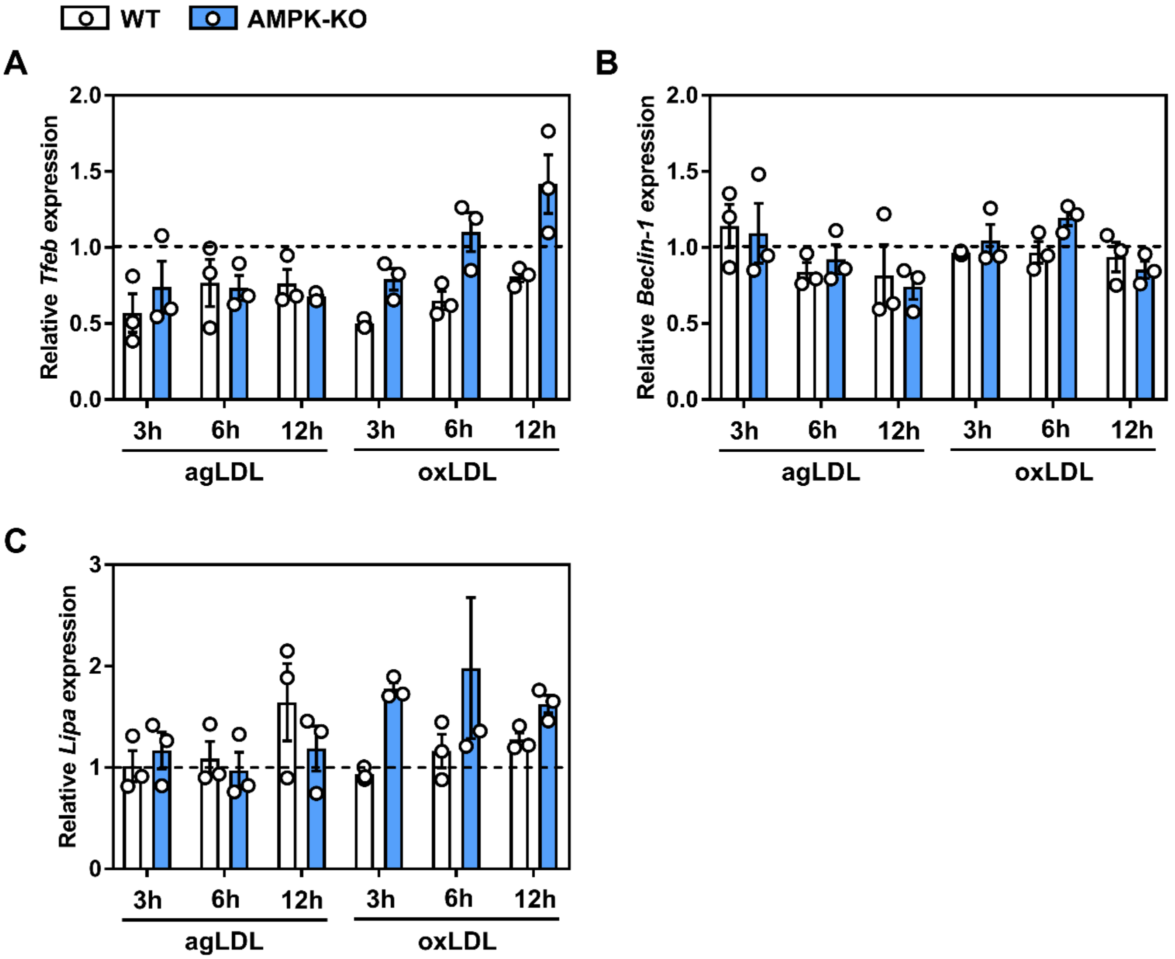

Supplementary Figure S4. Atherogenic lipoproteins uncouple macrophage AMPK's ability to regulate lysosomal-associated gene transcription. (A-C) Relative transcript expression for (A) *Tfeb*, (B) *Beclin-1* and *Lipa* (lysosomal acid lipase) in the presence of atherogenic lipoproteins agLDL and oxLDL (50 µg/mL) over time. Transcript expression was made relative to untreated WT macrophages and normalized to the average of *β actin* and *Tbp*. Data represent the mean ± SEM.

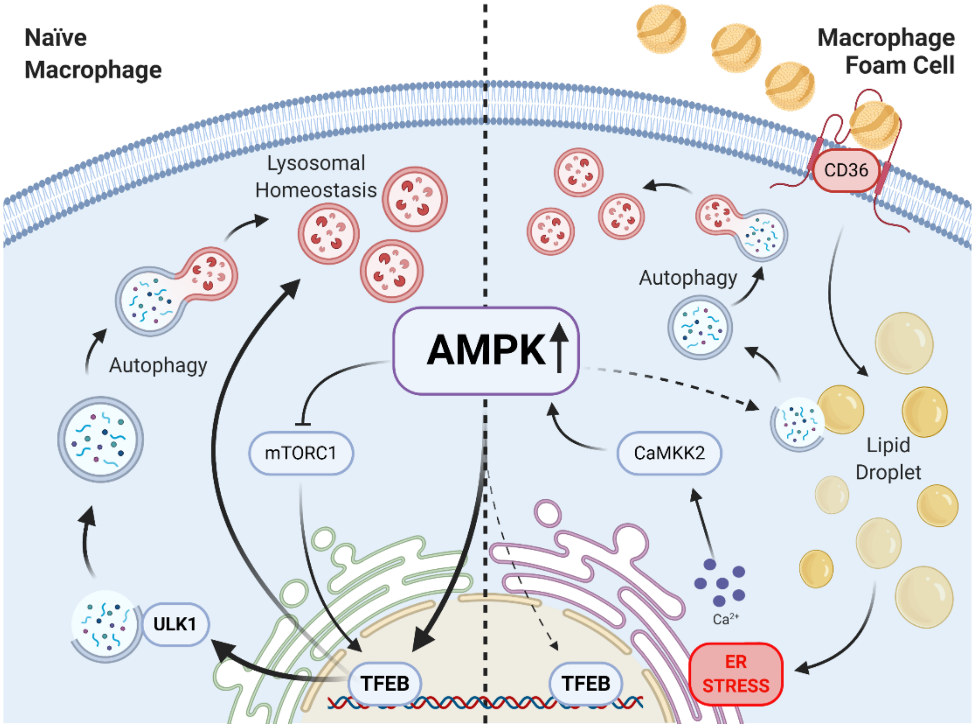

Supplementary Figure S5. Graphical abstract
